# To fight tuberculosis, fund basic research

**DOI:** 10.1371/journal.pbio.3000037

**Published:** 2018-09-25

**Authors:** Christoph Grundner

**Affiliations:** 1 Center for Infectious Disease Research, Seattle, Washington, United States of America; 2 Department of Global Health, University of Washington, Seattle, Washington, United States of America

## Abstract

Tuberculosis (TB) is now the leading cause of death from infectious disease. On September 26, 2018, the United Nations (UN) General Assembly holds its first high-level meeting on TB, a once-in-a-lifetime chance to commit governments around the world to redouble their TB control efforts. Here I share impressions from a preparatory meeting at the UN in June and make the case for basic research as a central component of any future TB control strategy. The pathogen that causes TB, *Mycobacterium tuberculosis*, is still largely a mystery. But if we do not understand the basic, fundamental workings of the pathogen, we cannot hope to develop 21st century interventions for the disease.

## Abstract 2

As the United Nations General Assembly meets for its first high-level meeting on tuberculosis (TB) on September 26, this Research Matters article urges the need to consider basic research as an essential component of any future anti-TB strategy.

Earlier this summer, I was among several hundred activists, officials, and other stakeholders who met to help shape the agenda of the upcoming UN General Assembly meeting on TB. We hope this meeting will draw the world’s heads of state to create a watershed moment in the fight against TB. And a watershed moment is desperately needed, because we are losing that fight. TB is now the world’s number one infectious killer [1], and during the time of the meeting alone, some 1,500 people died from TB. At the current rate, we will miss the World Health Organization’s 2035 TB elimination goal by around 150 years. We cannot change this trajectory without expanding our investments in basic research, as our current tools to prevent, diagnose, and treat TB are hopelessly outdated. This upcoming meeting is a chance to galvanize the global political will towards a redoubled effort to control TB and to support the science we need to do it. This may be the last such chance in a generation.

A first look at the several hundred participants gathered at the UN revealed just how long the reach of TB is: health ministers, members of parliaments, NGOs, doctors, and TB survivors from every corner of the world came to share their stories and implore their governments to act: A representative of the Canadian Inuit spoke about the destruction TB has wrought in parts of her community, where the TB incidence is 300 times higher than in the rest of Canada. Many spoke on behalf of marginalized communities such as refugees, miners, and prisoners for whom TB is exacerbated by poverty, exploitation, and close quarters. Some of the most wrenching stories were told by TB survivors, like the young Peruvian man whose TB cost him half his lung and his siblings’ education as the family struggled for years to pay for his treatment.

Several themes resonated throughout the day: far too many people go undiagnosed and untreated. In the Philippines, as many as 80 percent of people with TB do not get proper treatment, said Angelina Tan, a member of the Philippine parliament and the main author of her country’s landmark TB legislation. Her fight is personal, as her own mother’s TB cost her family its livelihood. Her story illustrates another recurring theme: controlling TB cannot stop at treating the sick, but also needs to help patients’ families and communities deal with the punishing economic consequences. But even those who do get treated often suffer from grueling side effects. It takes two years and nearly 15,000 pills to treat multidrug-resistant TB; many patients struggle with side effects that can make dying from TB seem like the better option.

While we need better implementation of existing tools to control TB, most are woefully outdated and inadequate—a stick in a gunfight, as one panelist put it. Most TB drugs predate man’s landing on the moon. The vaccine was first used in humans before movies had sound. And our main diagnostic takes several weeks during which patients can infect their whole family. The only thing that will help us develop effective diagnostics, drugs, and a vaccine, however, is basic research.

*Mycobacterium tuberculosis* has evolved resistance to every TB drug. And resistance to the newest TB drug, the first in 40 years, has already emerged during clinical trials. Just making new drugs will not break this cycle. Only treatments that target the very mechanisms of drug resistance and block the bacterium’s escape routes will prevent drug resistance. We cannot figure out how to make drugs that are resistant to resistance without basic research.

The current vaccine is not much of a vaccine, as most people who die of TB die vaccinated. Without a better understanding of the immune response to TB, the challenges of making an effective vaccine may be insurmountable. We need to understand when the immune system fails and when it succeeds against TB before we can engineer a response that truly protects.

Any effort to fight TB without a firm footing in basic research will inevitably fall short as the next drug-resistant strain emerges and the next vaccine fails. Better and sustained funding for basic science research must be a priority at the September UN General Assembly high-level meeting on the fight against tuberculosis. And basic research needs to be a parallel track to implementing the tools we already have. Without even considering the lives that could be saved, just the economic upside to every dollar spent on TB control is upwards of $40. Nothing about TB is easy, except for this calculation.

The entire meeting can be watched here: https://www.un.org/pga/72/2018/06/04/civil-society-hearing-on-the-fight-against-tuberculosis/.

A 6 minute long edited version can be watched here: https://www.youtube.com/watch?v=vwFfhFBkVxE&feature=youtu.be
